# Standardized Morphological Modeling and Simulation-Based Validation of a Novel Tibiotalar Fusion Implant

**DOI:** 10.3390/bioengineering12070705

**Published:** 2025-06-27

**Authors:** Chao-Wei Huang, Yu-Tzu Wang, Chi-An Chen, Chun-Li Lin

**Affiliations:** 1 Department of Biomedical Engineering, National Yang Ming Chiao Tung University, Hsinchu 300, Taiwan; josh.hcw@gmail.com; 2 Department of Orthopaedic Surgery, Taipei City Hospital, Taipei 103, Taiwan; 3 Department of Mechanical and Electro-Mechanical Engineering, TamKang University, New Taipei City 251, Taiwan; ytlwh@mail.tku.edu.tw (Y.-T.W.); doraemon781227@gmail.com (C.-A.C.); 4 Medical Device Innovation & Translation Center, National Yang Ming Chiao Tung University, Hsinchu 300, Taiwan

**Keywords:** tibiotalar joint, computed tomography, fusion implant, finite element analysis, contact

## Abstract

This study establishes a standardized geometric model of the tibiotalar joint based on anatomical morphology and validates its statistical representativeness. Using this model, a novel fusion implant was developed and evaluated for its biomechanical performance through nonlinear finite element (FE) analysis compared to traditional fixation methods. A morphological database of the tibiotalar joint was built using 30 computed tomography (CT) scans to determine key dimensional parameters, and a novel fusion implant was designed to match the joint’s natural curvature. FE analysis compared three fixation strategies: (1) the novel implant with an anterior plate, (2) the anterior plate alone, and (3) three compression screws. Biomechanical parameters, including joint contact area, micromotion, and stress distribution, were analyzed under simulated loading conditions. The novel implant achieved the highest joint contact area (95.0%) and lowest tibial micromotion (0.033 mm), significantly reducing stress concentration compared to anterior plate fixation (49.8% contact; 0.068 mm micromotion) and compression screws (78.2% contact; 0.355 mm micromotion). Constructing a standardized tibiotalar joint model with verified normal distribution is crucial for ensuring broad implant applicability. FE analysis demonstrated that the novel implant enhances joint contact, reduces micromotion, and optimizes stress distribution, offering a promising approach for improving surgical outcomes in tibiotalar joint fusion.

## 1. Introduction

Primary ankle osteoarthritis and traumatic osteoarthritis are commonly attributed to degeneration, dislocation, ligament injuries, or prolonged joint instability [1]. Advanced stages of ankle arthritis often result in the swelling and deformity of the tibiotalar joint, leading to pain and restricted mobility. Ankle arthrodesis (AA) is a widely adopted clinical intervention [2,3]. The procedure involves the debridement of necrotic joint cartilage followed by the fixation of the tibia and talus using three compression screws and an anterior plate or a lateral plate to achieve the joint fusion of the tibiotalar joint [2].

Studies have demonstrated that successful osseous fusion depends on achieving a large bone-to-bone contact area, increased compression at the fusion site, and the initial stability provided by rigid fixation [4,5,6]. Some studies have shown that anterior fixation plates exhibit superior fixation rigidity compared to compression screws, likely due to their enhanced ability to maintain the stability and contact area of the joint surfaces. When complications such as bone slippage or gaps between bones occur [7], the load-bearing contact area of the joint surface is reduced, resulting in stress concentration [3]. Such conditions can accelerate implant-associated fractures, nonunion [7,8,9,10], or limb malalignments (6.3%) [7,8], significantly impairing the outcomes of tibiotalar joint fixation and fusion.

The use of fusion implants that conform to the tibiotalar joint’s curvature and anatomical morphology can enhance fixation stability and prevent stress concentration. Maintaining maximum joint surface contact to optimize load-bearing areas remains critical in AA procedures [11]. Similar challenges are observed in the design of spinal cages and artificial intervertebral discs, where implants must closely match the vertebral endplate surface to minimize stress concentration and reduce the risk of subsidence. Studies have demonstrated that endplate-conforming spinal cages, designed based on the anatomical curvature of the endplate, can improve load transmission, enhance stability, and promote bone fusion [12]. Additionally, a study involving 138 patients measured the anatomical dimensions of the C4-C7 cervical vertebrae to design artificial cervical discs that match the endplate morphology, achieving uniform load distribution and mitigating subsidence risks [13,14]. These findings underscore the importance of implant geometry in maintaining contact area, increasing fixation stability, and facilitating bone fusion.

**Figure 1:**
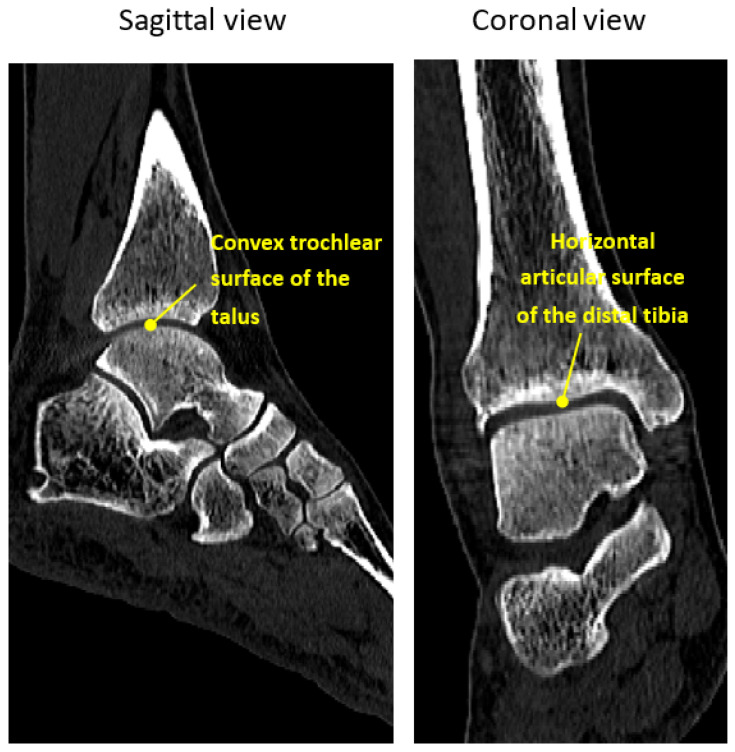
Sagittal and coronal views of the ankle CT image.

The curvature of the tibiotalar joint is particularly complex, involving the horizontal articular surface of the distal tibia, the medial malleolus, and the lateral malleolus of the fibula, which articulate with the convex trochlear surface of the talus (Figure 1). This unique anatomy allows for dorsiflexion and plantarflexion movements while providing joint stability. Anatomically conforming fusion implants can also help maintain the height of the tibiotalar joint, ensuring the affected limb remains consistent in length with the contralateral side, which is crucial for restoring normal postoperative gait. Despite these advantages, no reliable and effective implant design that conforms to the tibiotalar joint’s anatomy has been proposed in the literature.

This study aims to address this gap by developing a novel tibiotalar fusion implant based on the anatomical morphology of the joint. Using a dataset of 30 computed tomography (CT) scans, a comprehensive database of joint dimensions was constructed, and statistical analyses validated the anatomical plausibility of the joint’s geometry. Based on these dimensional data, a novel tibiotalar fusion implant was developed, and finite element (FE) analysis was performed to evaluate its biomechanical performance. The new implant was compared to anterior plate fixation and three compression screws. This study proposes that using an anatomically conforming fusion implant in conjunction with an anterior plate provides biomechanical advantages by reducing stress concentration on the joint surfaces, ensuring fixation stability and promoting effective bone fusion.

## 2. Materials and Methods

### 2.1. Establishment of the Tibiotalar Joint Morphological Parameter Database

**Figure 2:**
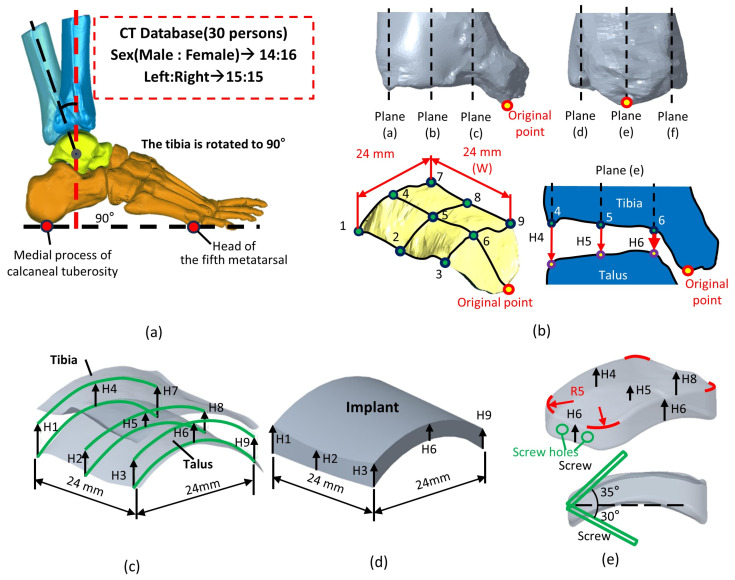
Methods for measuring the dimensions of the tibiotalar joint: (**a**) Collect 30 cases of CT images and reconstruct the tibiotalar joint model. (**b**) Define a method for measuring the spatial characteristics of the tibiotalar joint. (**c**) Identify nine measurement points within the tibiotalar joint space to serve as the basis for implant dimensions. (**d**) After measuring H1–H9, generate the implant surface contour. (**e**) Add an R5 mm fillet to the edges of the implant.

This study received approval from the Taipei City Hospital Research Ethics Committee for clinical research on ankle joint images (Project Title: The Morphological Analysis of Adult Knee & Ankle Joints and the Novel Implant Design, IRB No.: TCHIRB-11304022-E). Computed tomography (CT) scan images of 30 normal ankle joints (16 males, 14 females) were collected to establish a dimensional database (Mimics 22.0, Materialise NV, Leuven, Belgium). The dataset included 15 cases of left and 15 cases of right lower limb images (Figure 2a).

To measure the joint space, the tibia was aligned perpendicular to the plantar surface, ensuring a 90° angle between the tibia and the line connecting the head of the fifth metatarsal to the medial process of the calcaneal tuberosity (Figure 2a). This alignment was crucial for accurately assessing the tibiotalar joint space in a simulated standing position. In the coronal and sagittal sections of the inferior articular surface of the tibia, a central plane (plane (b,e)) was defined (Figure 2b). Additional planes (a,c,d,f) were offset by 12 mm on either side of the central plane. These six orthogonal planes intersected the inferior tibial articular surface, creating nine points where the distances between the tibia and talus (H1–H9) were measured, representing the spatial dimensions of the tibiotalar joint (Figure 2b,c).

After measuring the spatial dimensions (H1–H9) of the tibiotalar joint, the mean and standard deviation of each value were calculated. Subsequently, the absolute differences between each H value and the corresponding mean (H_ave._) were summed to yield a total difference value, V. A smaller value of V_sum_ indicates that the tibiotalar joint model closely matches the average surface morphology across the measurements. This suggests that the model is highly representative of the average dimensions of the tibiotalar joint [15].

V_sum_ of differences= ∣(H_ave._ − H_1_)∣ + … + ∣(H_ave._ − H_9_)∣

A smaller value of V_sum_, which means that the dimensions of the tibiotalar joint model closely approximate the mean value, served as a representative model.

Patient 30 (P30) can be considered a representative finite element (FE) model for the subsequent biomechanical analysis of the tibiotalar joint, as this patient exhibited the smallest V-value of 7.9. This indicates that the joint surface closely resembles the average morphology.

### 2.2. Verification of the Normal Distribution of Morphological Parameters of the Tibiotalar Joint

The dimensional database of the tibiotalar joint was primarily established using 30 sets of medical images of normal tibiotalar joints, measuring the distances between the tibia and talus (H1–H9) to characterize the surface morphology of the joint. This study employed the Kolmogorov–Smirnov (K-S) test to determine whether the data followed a normal distribution. The cumulative distribution function (CDF) of the sample distribution was compared to that of the normal distribution, and the maximum deviation between the two was calculated. The cumulative distribution function represents the cumulative probability that data points are less than or equal to a specific value x. The maximum deviation D between the sample distribution and the theoretical distribution was calculated using the following formula:

D = max ∣Fn(x) − F(x)∣

where D is the K-S statistic, representing the maximum difference between the sample and theoretical distributions. Fn(x) is the empirical cumulative distribution function (ECDF), reflecting the proportion of sample data less than or equal to x. F(x) is the theoretical cumulative distribution function, such as the cumulative probability of a normal distribution.

The D-value’s *p*-value was then calculated. When *p* > 0.05, it indicates no significant difference between the sample distribution and the normal distribution, suggesting that the data follow a normal distribution. Conversely, *p* ≤ 0.05 *p* indicates that the data deviate significantly from a normal distribution [16]. Additionally, a normal quantile–quantile plot (Q-Q) plot was used to standardize the data and compare them with a standard normal distribution.

Furthermore, to further validate the normal distribution characteristics of the 30 sets of medical images in the tibiotalar joint image database, standard deviation analysis was employed to assess normality. In a typical normal distribution, approximately 99.7% of the data should fall within three standard deviations of the mean.

### 2.3. A Novel Tibiotalar Joint Fusion Implant Design and Implant/Tibiotalar Joint Surface Morphology Deviation

The average values from H1 to H9 of the tibiotalar joint were used to define the height and shape of the standard fusion implant dimensions for the tibiotalar joint. The implant was designed as a 24 × 24 mm block, with its height tailored to the measured values from H1 to H9 (Creo Parametric v8.0, PTC, Needham, MA, USA). This height optimization generated a curved surface that conforms to the anatomy of the tibiotalar joint (Figure 2d). To ensure a smooth contour and facilitate implantation, a 5 mm radius fillet was added to the edges. For fixation, two screws were inserted through the implant at angles of 35° and 30° relative to the horizontal plane. One screw anchored the implant to the tibia, and the other secured it to the talus, ensuring stable fixation of the tibiotalar joint (Figure 2e).

To test the error between the standard fusion implant size and the random ankle size, a surface deviation analysis (Geomagic Studio, v12, Geomagic Inc., Morrisville, NC, USA) was conducted to evaluate the conformity between the implant and bone surfaces. The analysis included three tibiotalar joint medical images: one was from the original 30-set database and the other two were randomly selected from other set cases (Random1 and Random2).

**Figure 3:**
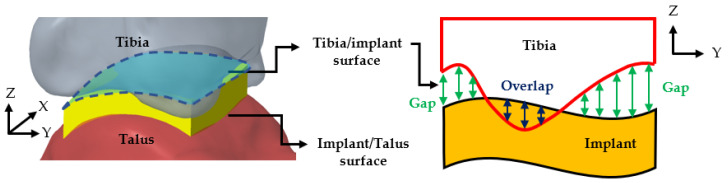
Surface deviation analysis simulating the conformity between tibiotalar joint surface and implant.

For each joint, 10,000 evenly distributed points were placed across the implant’s fitting surface (24 × 24 mm) to compare it with the corresponding bone surfaces. The deviation distances from each point to the tibial and talar surfaces were calculated, where positive values indicated a gap and negative values indicated overlap (Figure 3). An acceptable deviation threshold of 3 mm (the acceptable margin of error for surgery) was established, defining the level of conformity necessary for effective tibiotalar joint fusion reconstruction surgery.

### 2.4. Biomechanical Analysis of Three Tibiotalar Joint Fixations

**Figure 4:**
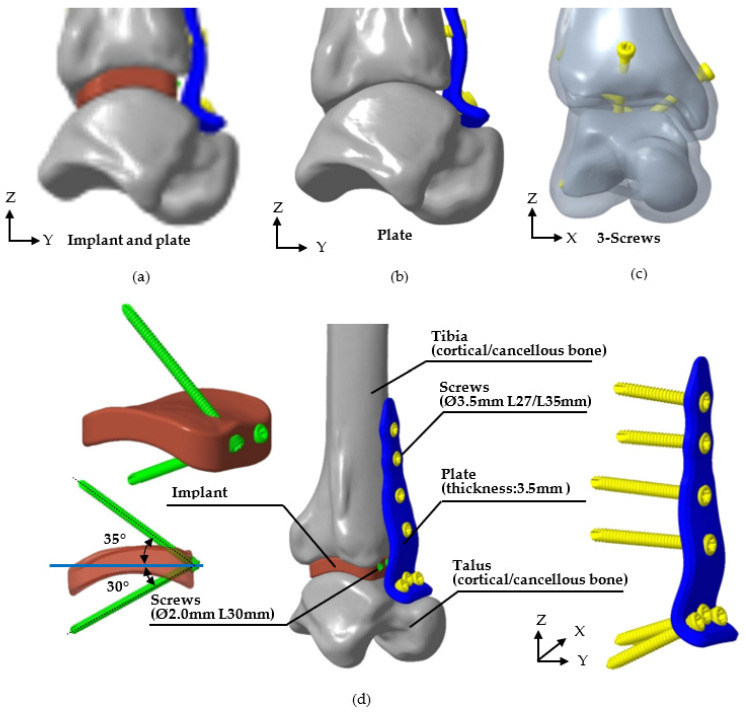
Three methods of tibiotalar joint fixation and the specifications of each corresponding implant. (**a**) The novel tibiotalar fusion implants combined with an anterior plate, (**b**) the anterior plate, (**c**) three compression screws, and (**d**) the specification of various implants.

This study utilized FE software (ANSYS 2023R2, ANSYS Inc., Canonsburg, PA, USA) to perform biomechanical analysis, verifying the stability of the novel fusion implant for tibiotalar joint fixation between the tibia and talus. The FE model was based on the P30 tibiotalar joint, which exhibited the smallest mean surface deviation in previous analyses, representing an average joint model. The bone model included both cortical and cancellous bone, and three fixation methods were simulated: (1) the novel tibiotalar joint fusion implant combined with an anterior plate, (2) the anterior plate alone, and (3) three compression screws. The number of screws used in each model is shown in Figure 4. All models were assigned isotropic and homogeneous material properties, with the relevant material characteristics detailed in Table 1.

**Figure 5:**
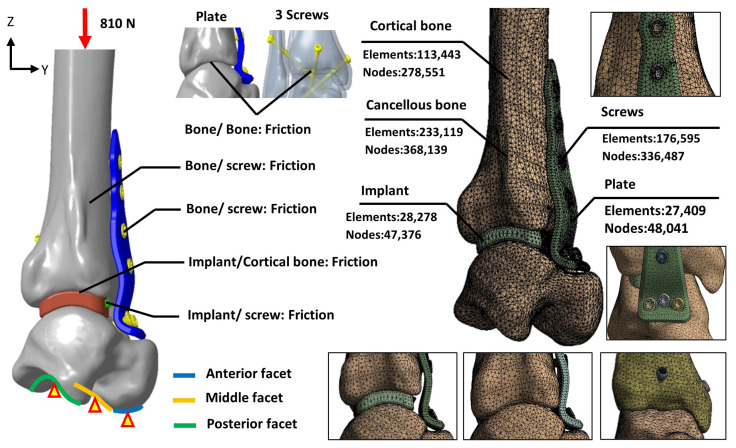
The loading, boundary conditions, and the mesh models of various fixation methods.

The mesh for each model was constructed using a free-meshing approach with 10-node tetrahedral elements. To prevent mesh size from affecting analysis results [17], a convergence study was performed using five different mesh sizes. The study monitored tibiotalar joint surface displacement across mesh sizes, and a threshold of less than 5% variation between analyses was used to confirm convergence [18]. Convergence results indicated that reducing the mesh size from 2 mm to 1 mm lowered the analysis error to 3.8%. Thus, a mesh size of 1 mm was selected for the analysis, with the final mesh configuration and element count shown in Figure 5.

**Table 1:** The material properties of the tibiotalar joint analysis model.

Material Properties	Elastic Modulus, E (MPa)	Poisson’s Ratio, ν	Mass Density (g/cm^3^)	References
Cortical bone	7300	0.3	1.845	[19]
Cancellous bone	1000	0.3	0.8	[20]
Implant	110,000	0.3	7.56	[21,22]
Plate				
Screw				

The biomechanical analysis simulated a single-leg stance, where the tibia bears 84.3% of the body weight [20,23]. The patient’s weight was assumed to be 98 kg, and a downward force of 810 N was applied to the proximal tibial surface to represent the load borne by the tibiotalar joint. Boundary conditions were defined by fixing the degrees of freedom (DOF = 0) at the anterior, middle, and posterior facets of the talus to stabilize the model. To simulate the immediate postoperative condition before bone ingrowth, the contact elements were used to mimic the adaption between bone–implant, bone–plate, and implant/plate–screw interfaces, and the coefficient of friction was set at 0.3, allowing for sliding resistance proportional to the applied force and permitting separation between objects (Figure 5).

The contact ranges of the joint surfaces were analyzed using contact elements to determine the outcomes under load. Results were categorized into four contact states: sticking (closed and sticking), sliding (closed with sliding), near (open but near contact), and far (open and not near contact). The numbers of sticking and sliding elements were quantified to calculate the percentage of contact area, indicating the ability of each implant to stabilize and maintain joint surface contact.

The micromotion of the tibial articular surface, reflecting the stability of implant fixation; the von Mises stress distribution on the tibiotalar joint surfaces and implants, identifying potential stress concentration areas; and the percentage of contact area on the joint surface, quantifying the implant’s ability to maintain joint contact and load distribution, were recorded in the FE analysis.

## 3. Results

### 3.1. Tibiotalar Joint Morphological Parameter Database

The V-values calculated from the database indicated that Patient 18 (P18) had the highest V-value of 27.70, reflecting a significant deviation in tibiotalar joint space dimensions from the average (Table 2). In contrast, Patient 30 (P30) exhibited the lowest V-value of 7.9, suggesting that the curvature of the tibiotalar joint in this case closely aligns with the average morphology. Consequently, the P30 joint was selected as a representative finite element (FE) model for tibiotalar joint reconstruction and was used in the biomechanical analysis. The average measurements of the H-dimensions ranged from 3.72 to 6.85 mm. These mean values provided the basis for designing the novel tibiotalar joint fusion implants, ensuring that the implants’ shape and dimensions conformed to the anatomical structure of the tibiotalar joint.

### 3.2. Verification of the Normal Distribution of Morphological Parameters of the Tibiotalar Joint

**Figure 6:**
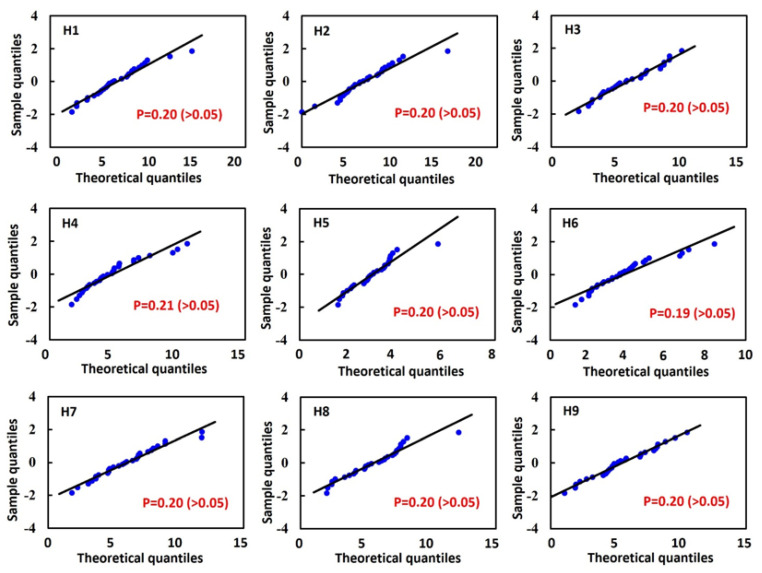
Results of the Kolmogorov–Smirnov (K-S) test: *p*-values for H1 to H9 and normal quantile–quantile (Q-Q) plots.

**Figure 7:**
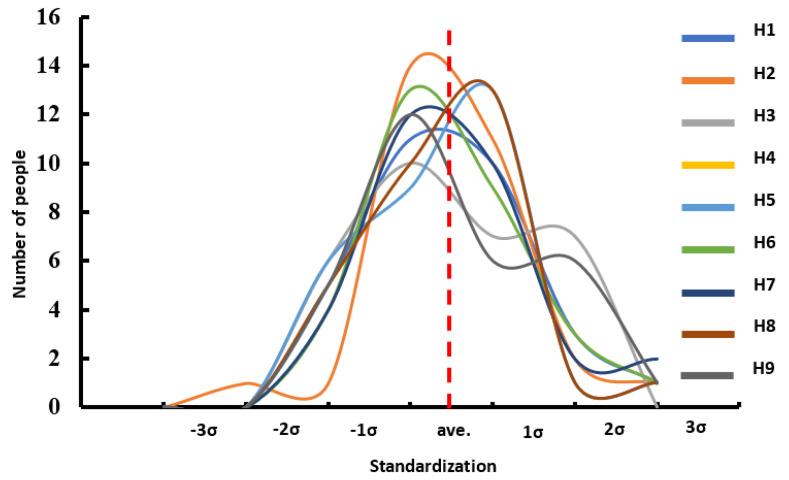
Result of the standardization of dimensional data in the tibiotalar joint space.

The K-S test for the normal distribution of H1 to H9 revealed that all *p*-values were greater than 0.05, indicating consistency between the sample distribution and a normal distribution. Additionally, by observing the Q-Q plot, which compares the sample data with the corresponding normal parent distribution, the data points for H1 to H9 were shown to be distributed close to the normal reference line. This confirms that the sample data follow a normal distribution. This finding validates the statistical representativeness and reliability of the database (Figure 6). In the standard deviation analysis, the results demonstrated that all data followed a normal distribution, with all samples falling within the 3σ range [24], indicating the absence of extreme values (Figure 7). This finding suggests that the surface morphology of the tibiotalar joint exhibits relatively low variability, with each H-value likely remaining within a consistent range. Consequently, the current image database can be considered representative.

### 3.3. Surface Morphology Deviation Between the Novel Tibiotalar Joint and the Implant

**Figure 8:**
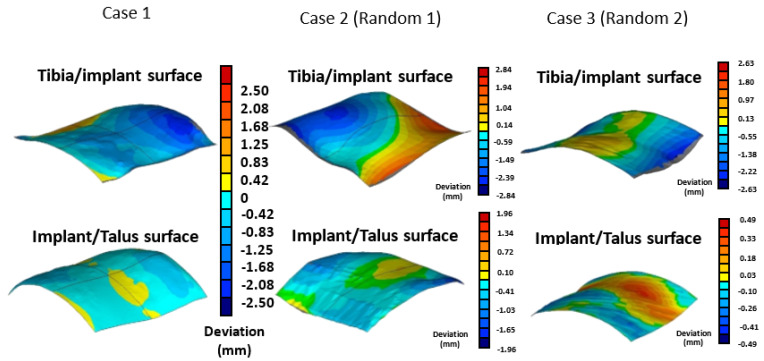
Verification of the conformity between the tibiotalar joint surface and the implant surface.

Deviation analysis indicates that the curvature of the tibiotalar joint fusion implant closely aligns with that of the representative model. The maximum deviation between the implant and the bone was −2.26 mm, the minimum deviation was 0.43 mm, and the average deviation ranged from 0.06 to 0.86 mm. For Random 1, the analysis revealed the largest deviation, with an average bias ranging from 0.16 to 1.14 mm and a maximum deviation of 2.84 mm. In contrast, Random 2 exhibited an average deviation ranging from 0.12 to 0.88 mm, with a maximum deviation of 2.63 mm (Figure 8). The deviation analysis results for all three groups were within the acceptance criteria established in this study, confirming that the tibiotalar joint fusion implants meet the requirements for use in the majority of patients.

### 3.4. Biomechanical Analysis of Three Tibiotalar Joint Fixations

**Figure 9:**
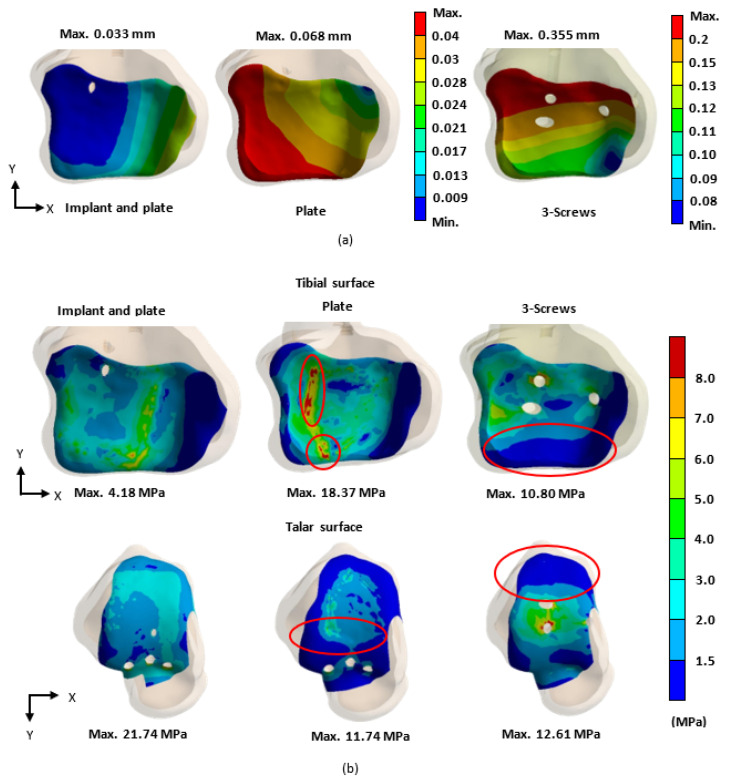
In the biomechanical analysis, the stability of the tibiotalar joint was evaluated based on (**a**) the micromotion of the tibial surface and (**b**) the stress distribution (von Mises stress) on the surfaces of the tibia and talus for each fixation type.

The biomechanical analysis of the three fixation methods revealed that the novel tibiotalar fusion implant combined with an anterior plate reduced tibial displacement to 0.033 mm. In comparison, fixation using an anterior plate alone and fixation with three compression screws resulted in tibial micromotion of 0.068 mm and 0.355 mm, respectively, indicating that these two methods provided relatively less stability (Figure 9a).

The stress distribution analysis of the tibiotalar joint surfaces showed that the novel tibiotalar fusion implant combined with plate fixation produced the lowest stress values among the three fixation methods. Stress concentrations were observed at the compression screw insertion sites in both the plate-only fixation and the three-screw fixation methods (Figure 9b).

**Figure 10:**
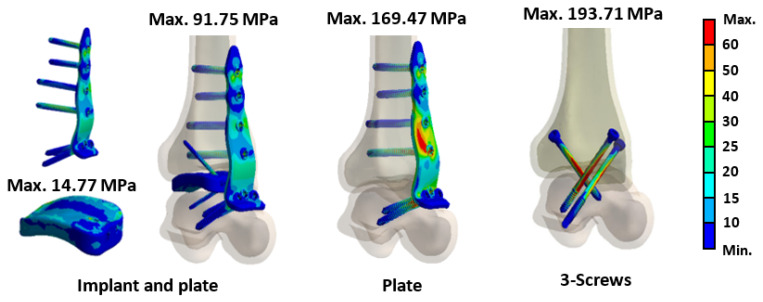
The results of von Mises stress for various implants.

Further stress analysis of the different fixation methods revealed that the stress for the novel tibiotalar fusion implant with a plate was 91.75 MPa. In contrast, the plate-only fixation and the three-screw fixation exhibited higher stress values, with the plate bearing 169.47 MPa and the screws bearing 193.71 MPa. These results indicate that the screws and plate absorbed a significant portion of the forces without effectively transmitting them to the tibia or talus, resulting in excessive stress concentration on the implants (Figure 10).

**Figure 11:**
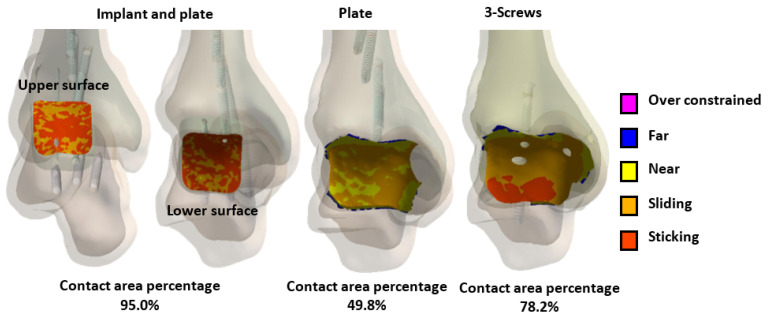
The percentage of joint contact area in the tibiotalar joint under different fixation methods after loading.

The contact area analysis of the joint surfaces for the three different fixation methods after tibiotalar joint fusion surgery revealed that the novel tibiotalar fusion implant combined with an anterior plate maintained the highest percentage of contact area (95.0%). This result indicates that this fixation method effectively preserves joint surface contact, reducing stress concentration. In contrast, the fixation method using only an anterior plate showed a significantly lower contact area of 49.8%, which may lead to joint instability and fusion failure. The fixation method with three compression screws achieved a contact area of 78.2%, ranking second in its ability to maintain joint surface contact area after the novel tibiotalar fusion implant combined with an anterior plate (Figure 11).

## 4. Discussion

For ankle arthrodesis (AA) surgery to achieve a fusion success rate of between 94% and 100%, it is essential to ensure high contact area bone alignment on the joint surfaces, increased compression at the fusion site, and initial stability through rigid fixation [4,5,6]. Therefore, the objective of this study is to develop a standardized tibiotalar fusion implant with anatomically representative morphological characteristics.

In this study, a statistical analysis from H1 to H9 was conducted using only 30 sets of CT images. To verify whether the numerical distribution adequately represents the population, standard deviation analysis and the Kolmogorov–Smirnov (K-S) test, which are commonly used for normal distribution assessments, were performed. The results showed that all values fell within three standard deviations above or below the mean, indicating that the data closely follow a normal distribution. Additionally, the K-S test and Q-Q plot results confirmed that the values adhered to a normal distribution, further supporting the validity of the reconstructed joint surfaces based on the 30 datasets.

Statistical analysis revealed that, among H1–H9, H5 exhibited the smallest value, located at the center of the tibiotalar joint. In contrast, the four measurement points around the periphery of the joint surface (H1/H3/H7/H9) showed the largest distances, indicating that the joint space resembles a concave lens shape. Additionally, the anterior joint space (H1/H2/H3) was slightly larger than the posterior space (H7/H8/H9), while the lateral joint space (H1/H4/H7) was greater than the medial space (H3/H6/H9), indicating that the tibiotalar joint is wider on the anterior and lateral sides. These results indicate that the tibia is positioned relative to the talus with a posterior and medial tilt. These anatomical characteristics of the tibiotalar joint align with observations in the literature regarding trends in tibiotalar joint space variations [25], further validating the measurement methods and results used in this study as reflective of actual joint anatomy. Based on these anatomical features, the tibiotalar joint fusion implant designed in this study also adopts a concave lens shape to better conform to the natural anatomy of the tibiotalar joint, enhancing its fit and stability.

To validate the usability of the reconstructed standard tibiotalar joint surfaces based on this database, they are expected to be applicable to the majority of patients undergoing tibiotalar joint fusion surgery. In the implant-to-joint surface deviation analysis, two sets of tibiotalar joint medical images were randomly selected, and the maximum deviation was found to be only 2.84 mm, which is within clinically acceptable limits. This result further confirms that the surface morphology of the tibiotalar joint fusion implant can accurately adapt to tibiotalar joints beyond those included in the database.

To understand the stability of the novel bone fusion implant designed using the standard tibiotalar joint surfaces proposed in this study, FE analysis was employed to investigate the biomechanical behavior of the tibiotalar joint following fusion. FE analysis is a well-established non-invasive tool for evaluating the biomechanical properties of various implants [20,26,27]. It simulates the geometry of the tibiotalar joint and the implant using mesh elements, incorporates appropriate material properties and physiological loads, and ultimately calculates the stress and strain on each structure to assess the stability of the tibiotalar joint [28,29]. The importance of FE analysis lies in its ability to quantify the biomechanical behavior of implants during the early postoperative phase, particularly before complete bone integration is achieved. It allows for the evaluation of implant-to-bone fixation stability and relative displacement [30]. Compared to animal studies or clinical trials, FE analysis offers a more precise quantification of early biomechanical characteristics, enabling the validation of the implant’s biomechanical behavior in relation to the anatomical structure of the tibiotalar joint [27].

In this study, the representative bone finite element (FE) model was reconstructed using P30, which exhibited the smallest deviation from the mean V-value, and three different implant configurations were analyzed. To validate the model’s reliability, convergence analysis [18] was performed to determine the optimal mesh size for the analysis model, and the results were compared with the relevant literature. This verification confirmed that the biomechanical analysis model and mechanical conditions aligned with the biomechanical characteristics of the tibiotalar joint.

Somberg A.M. conducted experiments using fresh-frozen human cadaver specimens of the tibiotalar joint, in which all muscles, ligaments, and articular cartilage were removed. The tibiotalar joint was then fixed using three standard cancellous bone screws with fixation points similar to those in the three-screw fixation group of the present study. In the referenced biomechanical tests, the talus was secured to a testing apparatus while a load fixture applied a downward force ranging from 0 to 525 N at a frequency of 1 Hz to the proximal tibia. The test was conducted over 500 loading cycles, with displacement values recorded at cycles 1, 10, 50, 100, and 500 to evaluate the stability of the tibiotalar joint fixation with three screws [26].

In this study, the biomechanical analysis of the tibiotalar joint was conducted under identical fixation and mechanical conditions. The results were compared with the displacement measurements from the initial loading tests in the literature. The biomechanical analysis indicated that the displacement of the tibiotalar joint in this study was 0.339 mm (339.3 μm), while the literature reported a displacement of 353.74 μm, resulting in a difference of approximately 4.08%. These findings demonstrate that the results of this study are comparable to those from the literature, indicating sufficient accuracy in the biomechanical analysis [31,32,33]. Furthermore, this validates that the newly developed tibiotalar joint fixation implants provide optimal stability (Table 3).

This study highlights the importance of selecting fixation methods that ensure proper load distribution and contact area conformity for joint surfaces. It is recommended that clinicians prioritize fixation methods with high conformity to the joint surface based on patient needs and postoperative stability requirements to improve surgical success rates [4,5,6]. The biomechanical analysis results revealed that, under load, the novel tibiotalar joint fusion implant achieved a high joint surface conformity of 95% (592.1 mm^2^). In comparison, the joint surface contact area was reduced to 49.8% (131.1 mm^2^) with plate fixation and to 78.2% (439.4 mm^2^) with three compression screws (Figure 11). By comparing the relationship between joint surface contact area and stress distribution under load, it becomes evident that maintaining the joint surface contact area is critical for reducing stress concentration and ensuring effective joint stabilization.

When the tibiotalar joint is fixed using an anterior plate alone, stress is typically concentrated on specific contact areas. This localized stress may increase the risk of bone damage and reduce fixation stability, while some contact areas may receive little to no contact stress, potentially leading to areas without any contact at all. Such conditions could negatively impact bone fusion outcomes in the future. In contrast, when the tibiotalar joint is fixed using three compression screws, stress is concentrated around the screws (Figure 9b). While this method maintains a greater contact area compared to the anterior plate alone, the posterior joint surface is nearly in a sliding state, receiving no contact stress. This results in even worse joint surface stress distribution and displacement compared to the anterior plate fixation, highlighting poor fixation stability [7,8,9,10]. The novel tibiotalar joint fixation implant developed in this study effectively facilitates contact between the tibial and talar surfaces, reducing uncertainty in joint surface contact. The biomechanical analysis further confirmed that the joint surface displacement was the lowest at 0.033 mm. Compared to the other two commonly used fixation methods, the novel tibiotalar joint fixation implant provides the most stable fixation effect (Figure 9a).

Although the current image database (30 cases) suggests that the anatomical morphology of the normal tibiotalar joint may lie within a limited and clinically definable range, expanding the dataset to include more diverse samples would enhance the robustness and applicability of the implant design. Furthermore, future research should explore the incorporation of a porous surface structure on the implant, as such modifications could enhance osseointegration within the tibiotalar joint, thereby improving both the fusion process and the overall fixation stability. In future work, the novel tibiotalar fusion implant will be physically fabricated and subjected to a series of validation procedures. The implant will be manufactured using selective laser melting (SLM) technology with medical-grade Ti6Al4V titanium alloy powder. Subsequently, mechanical testing and in vivo animal experiments will be conducted to quantify the implant’s structural strength, fatigue behavior, and biomechanical characteristics. These studies will further assess the safety, biocompatibility, and long-term fusion potential of the implant.

## 5. Conclusions

This study highlights the importance of reconstructing standard tibiotalar joint surfaces for the design of fusion implants. By leveraging a tibiotalar joint dimensional database and validating its statistical representativeness, the novel implant was shown to closely conform to joint anatomy, achieving superior biomechanical performance. Finite element analysis demonstrated that the implant maintained the highest joint surface contact area (95%) and the lowest displacement (0.033 mm), significantly reducing stress concentration compared to traditional fixation methods. These findings underscore the critical role of anatomically accurate joint reconstruction and biomechanical evaluation in advancing implant design for tibiotalar joint fusion.

## Figures and Tables

**Table 2:** Measurement results of the tibiotalar joint dimensions.

Case	Height									V_sum of differences_
	H1	H2	H3	H4	H5	H6	H7	H8	H9	
1	5.84	5.26	5.15	5.35	4.29	4.18	7.67	6.49	8.06	9.47
2	6.06	9.27	8.36	2.8	3.44	3.55	4.57	2.39	5.74	13.59
3	8.35	5.44	8.63	10.55	3.51	4.26	5.42	4.09	4.83	14.48
4	5.25	5.86	4.87	3.96	3.33	3.56	6.95	7.88	4.31	8.98
5	8.29	8.16	7.18	5.32	7.08	3.71	8.99	7.3	5.35	13.06
6	9.68	5.95	5.80	9.44	3.98	4.03	7.94	4.19	4.22	13.12
7	2.72	3.65	7.25	2.48	4.18	3.18	3.36	4.91	4.42	16.10
8	4.47	3.92	3.17	4.89	4.35	8.4	7.04	6.19	6.85	15.84
9	7.31	7.69	6.85	5.29	3.49	4.35	3.09	3.39	4.67	10.01
10	2.74	4.64	2.86	3.82	2.69	3.16	6.76	5.18	8.22	15.42
11	7.81	6.96	6.95	2.91	2.79	4.87	3.88	6.68	6.9	11.15
12	14.52	9.96	8.4	7.68	4.76	2.74	3.65	6.01	4.54	22.41
13	8.58	6.73	6.19	9.81	4.56	7.07	6.92	2.41	3.22	17.73
14	8.05	8.28	8.65	6.44	3.8	4.77	9.00	8.19	8.17	16.63
15	5.96	6.32	4.01	6.45	3.66	6.74	11.78	12.14	7.91	22.20
16	9.09	8.9	9.07	4.12	4.52	3.81	4.66	7.19	4.74	13.17
17	5.7	8.16	7.34	2.06	2.03	2.41	6.84	7.68	10.47	17.88
18	9.38	14.92	10	4.95	3.28	2.1	2.26	2.66	1.94	27.70
19	12.25	10.36	9.04	4.76	4.36	6.63	1.84	3.75	2.24	25.39
20	7.31	4.76	2.11	4.81	4.62	2.65	4.65	7.69	5.18	11.81
21	9.95	0	3.91	3.57	4.99	4.09	5.00	4.99	6.92	17.53
22	5.46	6.74	4.39	3.04	3.58	2.36	6.48	6.41	9.55	11.82
23	7.95	4.88	3.07	1.67	1.97	1.6	3.67	7.04	5.8	16.73
24	2.23	1.34	5.25	3.42	2.19	2.15	5.72	4.25	4.02	18.15
25	6.55	8.5	4.99	2.29	2.6	1.99	8.42	2.07	1.88	18.25
26	3.82	5.26	5.72	3.9	4.65	2.97	4.71	7.39	7.23	11.94
27	3.78	4.41	3.78	2.57	2.24	1.27	5.87	2.00	1.07	21.73
28	5.1	3.94	4.68	4.43	2.43	1.96	11.81	7.56	8.8	19.92
29	4.87	4.22	3.71	6.8	3.77	3.39	6.02	4.95	2.76	12.29
30	6.35	7.85	6.84	4.93	4.6	5.06	8.08	5.43	4.88	7.90
avg.	6.85	6.41	5.94	4.82	3.72	3.77	6.10	5.62	5.50	Implants Body Size (average)
Sd.	2.78	2.88	2.16	2.27	1.10	1.71	2.47	2.29	2.39	

**Table 3:** The comparison between the analytical data of this study and the biomechanical test data from the literature.

Analytical Model Validation				
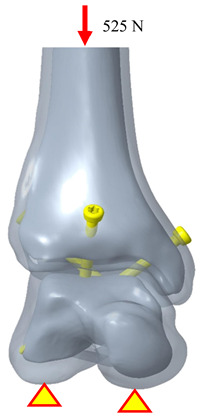	Micromotion	Finite element analysis (this study)	0.339 mm (339.3 μm)	
		Validation value (biomechanical test)	353.74 μm	[18]
		Difference (%)	4.08%	
	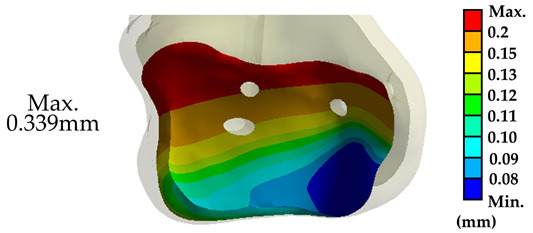			

## Data Availability

The original contributions presented in this study are included in the article. Further inquiries can be directed to the corresponding author.
